# Antibiotic‐Induced Gut Dysbiosis Modulates Alzheimer's Disease‐Associated Gene Expression and Protein Aggregation in 3xTg‐AD Mice via the Gut–Brain Axis

**DOI:** 10.1002/brb3.70946

**Published:** 2025-10-10

**Authors:** Edward Jenner Tettevi, David Larbi Simpong, Mahmoud Maina, Samuel Adjei, Elias Asuming‐Brempong, Mike Y. Osei‐Atweneboana, Augustine Ocloo

**Affiliations:** ^1^ Department of Biochemistry, Cell and Molecular Biology, School of Biological Science, Volta Road University of Ghana Accra Ghana; ^2^ West African Centre For Cell Biology of Infectious Pathogens, School of Biological Science, Volta Road University of Ghana Accra Ghana; ^3^ Council For Scientific and Industrial Research‐Water Research Institute, Biomedical and Public Health Research Unit, Agostinho Neto Road, Council Close, Airport Residential Accra Ghana; ^4^ Department of Medical Laboratory Sciences, College of Health and Allied Sciences University of Cape Coast Cape Coast Ghana; ^5^ Serpell Laboratory, Sussex Neuroscience, School of Life Sciences University of Sussex Brighton UK; ^6^ Department of Animal Experimentation, Noguchi Memorial Institute for Medical Research, College of Health Sciences University of Ghana Accra Ghana; ^7^ CSIR‐College of Science and Technology, second CSIR Close, Behind Lancaster, Airport Residential Accra Ghana

**Keywords:** 3xTg‐AD mice, cholinergic dysfunction, gut–brain axis, neurodegenerative

## Abstract

**Introduction:**

Alzheimer's disease (AD) is a progressive neurodegenerative disorder that poses a major global health challenge due to its increasing prevalence and lack of effective treatments. Emerging evidence suggests the gut–brain axis may play a pivotal role in AD pathogenesis. However, causal links between dysbiosis and late‐stage AD pathology remain unclear.

**Methods:**

This study evaluated the effects of antibiotic‐induced gut dysbiosis in aged 3xTg‐AD mice (46–48 weeks). Female mice were randomly assigned to control or treatment groups and administered a broad‐spectrum antibiotic cocktail (ampicillin, vancomycin, and neomycin) for 14 days. Behavioral tests (Y‐maze, elevated plus maze) were performed to assess cognitive and anxiety‐like behaviors. Gut microbiota composition was assessed via 16S rRNA qPCR. Gene expression of Acetylcholinesterase (AChE), Butyrylcholinesterase (BChE), and Tumor Necrosis Factor‐Alpha (TNF‐α) was analyzed via qRT‐PCR, and cerebral amyloid‐β_1–42_ and tau protein levels were quantified by ELISA.

**Results:**

Antibiotic treatment induced significant dysbiosis, with > 90% reduction in Firmicutes and Bacteroidetes. Dysbiotic mice displayed impaired spatial working memory, heightened anxiety‐like behavior, and reduced locomotor activity. Molecular analyses revealed region‐specific dysregulation of cholinergic genes: AChE was upregulated in the hippocampus but downregulated in the cortex, while BChE showed the opposite trend. TNF‐α was significantly elevated in both regions, indicating neuroinflammation. Dysbiosis also led to increased brain levels of amyloid‐β_1–42_ and tau.

**Conclusion:**

Gut microbiome disruption exacerbates late‐stage AD pathology, driving cognitive deficits, neuroinflammation, and hallmark protein aggregation. These findings support the gut–brain axis as a critical modulator of AD and highlight the microbiome as a potential therapeutic target.

## Graphical abstract

Antibiotic‐induced gut dysbiosis accelerates AD pathology via neuroinflammation, cholinergic enzyme dysregulation, and protein aggregation in the 3xTg‐AD mice (*n* = 5)

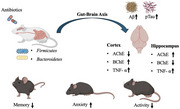

## 1 Introduction

Alzheimer's disease (AD) represents the most prevalent neurodegenerative disorder globally, characterized by progressive cognitive decline and distinctive neuropathological hallmarks including amyloid‐beta (Aβ) plaques, neurofibrillary tau tangles, and chronic neuroinflammation (Alzheimer 1906; Alzheimer et al. 1995; Adamu et al. 2024; Bertsch et al. 2023). Despite extensive research, the complex etiology of AD remains incompletely understood, with emerging evidence implicating the gut–brain axis as a critical modulator of neurodegeneration (Nasb et al. 2023).

The gut–brain axis encompasses bidirectional communication networks integrating neural, endocrine, immune, and metabolic pathways that connect the central nervous system with the enteric nervous system (Nasb et al. 2023; Alonso‐García et al. 2021). Within this framework, the gut microbiome‐comprising trillions of microorganisms residing in the gastrointestinal tract‐has emerged as a key regulator of brain homeostasis and neurological function (Anand et al. 2022; García‐Cabrerizo et al. 2020).

Accumulating evidence demonstrates that gut microbiome dysbiosis‐defined as pathological alterations in microbial community composition and diversity‐correlates with AD progression and cognitive decline (Nguyen et al. 2023; Sheng et al. 2023; Dağdeviren and Bozcal 2025). Mechanistically, dysbiosis influences neurodegeneration through multiple pathways (Zheng et al. 2023; Liu et al. 2023).

The cholinergic system plays a fundamental role in cognitive processes, with cholinergic dysfunction representing a well‐established hallmark of AD pathology (Hampel et al. 2018; Jabir et al. 2018; Spinelli et al. 2025). Acetylcholinesterase (AChE) and butyrylcholinesterase (BChE), the primary enzymes responsible for acetylcholine degradation, exhibit dysregulated activity in AD brains, contributing to cognitive impairment (Berson and Soreq 2010). Emerging evidence suggests that gut microbiome‐derived metabolites can modulate cholinergic enzyme expression and activity, potentially linking intestinal dysbiosis to cognitive dysfunction.

Despite growing recognition of the gut–brain axis in neurodegeneration, the causal relationship between gut dysbiosis and late‐stage AD pathology remains poorly defined. Most previous studies have focused on early‐stage disease models or correlative associations, leaving critical mechanistic gaps in our understanding of how gut microbiome disruption influences established AD pathology.

We hypothesized that antibiotic‐induced gut dysbiosis would exacerbate molecular and behavioral hallmarks of AD in aged 3xTg‐AD mice through dysregulation of cholinergic pathways, enhanced neuroinflammation, and accelerated protein aggregation. To test this hypothesis, we employed a well‐characterized triple transgenic AD mouse model at advanced disease stages (46–48 weeks) to investigate: (i) causal impact of dysbiosis on cognitive and anxiety‐related behaviors; (ii) region‐specific alterations in cholinergic enzyme gene expression (AChE, BChE); (iii) neuroinflammatory responses as measured by tumor necrosis factor‐alpha (TNF‐α) expression in specific brain regions; and (iv) changes in AD‐associated protein aggregation (Aβ_1–42_, total tau). This study addresses a critical knowledge gap by establishing causal mechanistic links between gut dysbiosis and AD progression in a clinically relevant late‐stage disease model. In this study, we focused on the hippocampus and cortex–brain regions critically involved in memory, cognition, and AD‐related pathology‐due to their established roles in cholinergic signaling, neuroinflammatory processes, and protein aggregation in AD (Câmara 2020; Braak and Braak 1991; Goschorska et al. 2018; Xing et al. 2025).

## 2 Methods

### 2.1 Experimental Design

This study employed a randomized controlled experimental design using female 3xTg‐AD mice to investigate the effects of antibiotic‐induced gut dysbiosis on AD‐related molecular and behavioral parameters. The experimental timeline included a 14‐day antibiotic treatment period, followed by behavioral assessments and molecular analyses.

### 2.2 Animals and Ethical Considerations

Breeding stock of 3xTg‐AD mice (Tg[APPSwe, tauP301L]1Lfa Psen1TM 1Mpm/Mmjax) was obtained from Jackson Laboratory (Bar Harbor, ME, USA) at 6 weeks of age and acclimatized for 30 days, and the colony was subsequently expanded at the Department of Animal Experimentation, Noguchi Memorial Institute for Medical Research, University of Ghana—Legon. For this study, female mice aged 46–48 weeks were selected based on established literature demonstrating that females exhibit more robust AD pathology and superior survival rates relative to males at advanced ages (Torres‐Lista et al. 2017; Muntsant et al. 2021; Judd, Winslow et al. 2024). This age range corresponds to late‐stage AD pathology, characterized by established amyloid and tau pathology with significant cognitive deficits (Oddo et al. 2003).

The animals were group‐housed (five per cage) during the study period, in ventilated steel cages (36 × 20 × 14 cm) containing autoclaved saw‐dust bedding. Environmental parameters were strictly controlled: 12:12 h light/dark cycle (lights on 7:00 a.m.), temperature 22–25°C, and relative humidity range between 50%–70% (Rhodes et al. 2004). Animals received ad libitum access to standard rodent chow (AGRIFEEDS; 17% protein, 3.68% fat, 3.35% fiber) and autoclaved water.

Animals were randomly assigned to experimental groups. The Control Group (*n* = 5) received a vehicle treatment consisting of sterile double‐distilled water, while the Dysbiosis Group (*n* = 5) was treated with an antibiotic cocktail.

All experimental procedures were conducted in accordance with international guidelines for laboratory animal care and use. Ethical approval was obtained from the Council for Scientific and Industrial Research—Animal Care and Use Committee (Protocol Number: CSIR009/2023).

### 2.3 Gut Microbiome Dysbiosis Induction

Gut microbiome dysbiosis was induced using a validated broad‐spectrum antibiotic cocktail, adapted from established protocols (Yang et al. 2015; P. Wang et al. 2021; Fröhlich et al. 2016). The treatment consisted of ampicillin (Pokupharma Limited, Ghana) at a final concentration of 5 mg/mL, vancomycin (Avet Pharmaceuticals, USA) at 2.5 mg/mL, and neomycin (Advancare Pharma, USA) at 5 mg/mL.

Fresh antibiotic solutions were prepared daily in sterile double‐distilled water and stored in light‐protected containers at 4°C. Treatments were administered via oral gavage (200 µL volume) once daily for 14 consecutive days between 8:00 a.m. and 10:00 a.m. to minimize circadian variability. Control animals received equivalent volumes of sterile double‐distilled water under identical conditions.

### 2.4 Gut Microbiome Analysis

Fecal samples were collected 48 h posttreatment completion to capture peak dysbiosis effects while avoiding acute antibiotic interference. Collection occurred between 08:00 a.m. and 09:00 a.m. to minimize circadian variations. Fresh fecal pellets were directly collected into sterile 2 mL Eppendorf tubes, weighed to 100 mg precision, and immediately preserved in DNA/RNA Shield solution (Zymo Research, USA). Samples were placed on ice and stored at −80°C until analysis.

Genomic DNA was extracted using the DNeasy Blood and Tissue Kit (Qiagen, Germany) following the manufacturer's protocols with modifications. Samples were treated with 1% w/v polyvinylpolypyrrolidone (PVPP; Sigma‐Aldrich, Germany) during lysis to remove PCR inhibitors. DNA concentration and purity were assessed using NanoDrop One spectrophotometry (Thermo Fisher Scientific, USA).

Microbial composition and diversity were analyzed using quantitative PCR (qPCR) targeting the 16S rRNA, the gold‐standard genetic marker for microbial identification‐with taxon‐specific primers detailed in Table 1 (Yang et al. 2015; Yadav et al. 2018). Reactions were performed in triplicate using the Bio‐Rad CFX96 Touch system (BioRad, Germany) under standardized conditions: initial denaturation at 95°C for 5 min, followed by 40 cycles of 95°C for 15 s and 60°C for 45 s. Cycle threshold (Ct) values were normalized to universal bacterial 16S rRNA primers (27F/1525R), and relative abundances calculated using the 2^(−ΔΔCt)^ method.

**TABLE 1:** 16S rRNA gene‐targeted group‐specific primers used in this study.

**Target group**	**Primer**	**Sequence (5′–3′)**
*Bacteroidetes*	Bac960F	GTTTAATTCGATGATACGCGAG
	Bac1100R	TTAASCCGACACCTCACGG
*Firmicutes*	Firm934F	GGAGYATGTGGTTTAATTCGAAGCA
	Firm1060R	AGCTGACGACAACCATGCAC
*Actinobacteria*	Act664F	TGTAGCGGTGGAATGCGC
	Act941R	AATTAAGCCACATGCTCCGCT
*Candidatus* Saccharibacteria	Sac1031F	AAGAGAACTGTGCCTTCGG
	Sac1218R	GCGTAAGGGAAATACTGACC
*Deferribacteres*	Defer1115F	CTATTTCCAGTTGCTAACGG
	Defer1265R	GAGHTGCTTCCCTCTGATTATG
*Verrucomicrobia*	Ver1165F	TCAKGTCAGTATGGCCCTTAT
	Ver1263R	CAGTTTTYAGGATTTCCTCCGCC
*Tenericutes*	Ten662F	ATGTGTAGCGGTAAAATGCGTAA
	Ten862R	CMTACTTGCGTACGTACTACT
*Betaproteobacteria*	Beta979F	AACGCGAAAAACCTTACCTACC
	Beta1130R	TGCCCTTTCGTAGCAACTAGTG
*Epsilonproteobacteria*	Epsilon940F	TAGGCTTGACATTGATAGAATC
	Epsilon1129R	CTTACGAAGGCAGTCTCCTTA
*Delta*‐and *Gammaproteobacteria*	Gamma877F	GCTAACGCATTAAGTRYCCCG
	Gamma1066R	GCCATGCRGCACCTGTCT
Universal	926F	AAACTCAAAKGAATTGACGG
	1062R	CTCACRRCACGAGCTGAC
Bacterial 16S rRNA	27F	AGAGTTTGATCCTGGCTCAG
	1525R	AAGGAGGTGWTCCARCC

### 2.5 Behavioral Assessments

Behavioral assessments were conducted 72 h post‐antibiotic treatment between 08:00 a.m. and 11:00 a.m. under standardized environmental conditions (ambient temperature 22°C–25°C, illumination 150 lux). All apparatuses were thoroughly cleaned with 70% ethanol between subjects to eliminate olfactory cues.

The Y‐maze consisted of three identical arms (30 cm length × 10 cm width × 15 cm height) (Garcia and Esquivel 2018) constructed from white Alucobond material and arranged at 120° angles. Mice with fewer than five total arm entries were excluded.

Animals were habituated to the testing room for 60 min before assessment, in individual cages. Each mouse was placed at the distal end of the starting arm and allowed free exploration for 5 min. Behavior was recorded using overhead digital cameras. Arm entries were scored when all four paws entered an arm. Spontaneous alternation percentage was calculated as: (Number of alternations/(Total arm entries − 2)) × 100, where alternation was defined as consecutive entries into three different arms (Garcia and Esquivel 2018).

During the training phase, one arm was blocked for 8 min while mice explored the two accessible arms. After a 1‐h delay, the barrier was removed and mice were placed in the center with access to all three arms for 5 min. Time spent in the previously blocked “novel” arm was compared to “starting” and “familiar” arms (Kraeuter et al. 2019).

The EPM consisted of two open arms (45 × 10 cm) and two enclosed arms (45 × 10 × 30 cm) extending from a central platform (10 × 10 cm), elevated 60 cm above floor level (Jürgenson et al. 2010).

Mice were placed in the center facing an open arm and recorded for 5 min. Behavioral parameters included: (i) time spent in open versus closed arms, (ii) total distance traveled, and (iii) exploratory patterns analyzed. Recorded data were analyzed using ToxTrac software v2.61 (Rodriguez et al. 2018; Harini et al. 2024; Souto‐Maior et al. 2011). To maintain consistency and reduce olfactory cues, the maze was cleaned with 70% ethanol after each mouse was tested, minimizing potential bias (Leo and Pamplona 2014). Mice performing fewer than four entries into different arms were excluded from the analysis.

### 2.6 Molecular Analyses

Brain tissue harvesting was done as previously described by Jaszczyk et al. (2022) with slight modification (Jaszczyk et al. 2022). Following behavioral assessments, animals were euthanized via isoflurane overdose. The head of each animal was disinfected by spraying 70% ethanol to minimize external contamination. A midline incision along the scalp was made using sterile surgical scissors, exposing the skull. The sides of the skull were then cut open using surgical scissors to expose the brain. Care was taken to prevent damage to the brain during this process. Brains were rapidly extracted and rinsed with ice‐cold 1× PBS, then placed on dry‐ice for dissection. Using a surgical scalpel, each brain was carefully divided into left and right hemispheres. The left hemisphere was allocated for gene expression analysis, while the right hemisphere was used for protein quantification. Under stereomicroscopic guidance, the hippocampus (CA1–CA3) and cortex (neocortex) were micro‐dissected and immediately preserved in DNA/RNA Shield solution (Zymo Research, USA) in 1.5 mL Eppendorf tubes and stored in an iced Eppendorf tube rack at −20°C.

Total RNA was extracted using TRI reagent (Ahmad et al. 2025) following proteinase K (Bhardwaj et al. 2025) treatment and purified using Direct‐zol RNA MiniPrep Kits (Zymo Research, USA). RNA quality and concentration were assessed via NanoDrop One Microvolume UV–vis Spectrophotometer (Thermo Fisher Scientific, USA). Complementary DNA (cDNA) synthesis (Özgür et al. 2023) was performed using 1 µg total RNA with LunaScript RT SuperMix (NEB, USA) according to the manufacturer's protocols.

Gene expression analysis was performed using Luna Universal qPCR Master Mix (NEB, USA) on a Bio‐Rad CFX96 Touch system. Reactions (20 µL) contained cDNA template (3 µL), gene‐specific primers (0.5 µL each), qPCR master mix (10 µL), and nuclease‐free water (6 µL). Thermal cycling conditions: reverse transcription at 50°C for 10 min, initial denaturation at 95°C for 5 min, followed by 40 cycles of 95°C for 15 s and 60°C for 45 s. Target genes included AChE, BChE, and TNF‐α, with GAPDH (Coulson et al. 2008) as the reference gene (Table 2).

**TABLE 2:** Target gene specific primers for differential gene expression analysis.

Target Gene	Primer (5'‐3')	Reference
*AChE*	**F** = CCTGGATCCCTCGCTGAA	Lestaevel et al. (2011)
	**R** = CCTGTGCGGGCAAAATTG	
*BuChE*	**F** = GTTTTCTGCAGTGAGTGACAGGTATT	Lestaevel et al. (2011)
	**R** = CAGAATCTGGATGCTGAAGTAGATAA	
*TNF‐α*	**F** = GCACAGAAAGCATGATCCG	X. Wang et al. (2022)
	**R** = TGAGTGTGAGGGTCTGGGC	
*GAPDH*	**F** = GTTGTCTCCTGCGACTTCA	Lee et al. (2019)
	**R** = GGTGGTCCAGGGTTTCTTA	

Abbreviations: AChE, acetylcholinesterase; BuChE, butyrylcholinesterase; GAPDH, glyceraldehyde‐3‐phosphate dehydrogenase; TNF‐α, tumor necrosis factor‐alpha.

### 2.7 ELISA Detection

Brain tissue homogenates were prepared from right hemisphere samples. Tissue (100 mg) was homogenized in 1 mL ice‐cold 1× PBS and stored at −20°C overnight to disrupt cell membranes. Two freeze‐thaw cycles were then performed to further disrupt the cell membranes, and centrifuged to obtain supernatants.

Human‐specific Aβ_1–42_ and tau kit were used to quantify the transgenes encoded in the 3xTg‐AD mice. Total Aβ_1–42_ levels were measured using the Human Amyloid Beta Peptide 1–42 ELISA Kit (Biomatik Corporation, CA; ECK32566) following the manufacturer's protocol with some modifications. Samples and standards were incubated at 37°C for 120 min, followed by biotinylated antibody detection and streptavidin‐HRP conjugation. Colorimetric development was measured at 450 nm using SpectraMax M5 spectrophotometry (Molecular Devices, USA).

Tau protein levels were determined using the Human MAPT ELISA Kit (Biomatik Corporation, CA; EKF57422). Samples were incubated on pre‐coated plates for 120 min at room temperature, followed by biotin‐labeled antibody incubation (60 min) and HRP‐streptavidin conjugate treatment (30 min). TMB substrate development was stopped after 20 min at 37°C, and absorbance was measured at 450 nm using SpectraMax M5 spectrophotometry.

### 2.8 Statistical Analysis

Sample size was determined based on previous studies using 3xTg‐AD mice with similar experimental paradigms, providing adequate power (> 80%) to detect biologically meaningful differences with *α* = 0.05.

qPCR data were analyzed using the 2^(−ΔΔCt)^ method with GAPDH normalization using qRAT software v0.2.0. ELISA data were fitted using 4‐parameter logistic (4PL) modeling with GainData software. Statistical analyses and plots were performed using GraphPad Prism v10.0. Between‐group comparisons utilized Student's *t*‐test, with *p* < 0.05 considered significant. Data is presented as mean ± standard error of mean (SEM).

## 3 Results

### 3.1 Confirmation of Antibiotic‐Induced Gut Microbiome Dysbiosis

Quantitative analysis of 16S rRNA gene copy numbers demonstrated significant alterations in gut microbial community structure following antibiotic treatment (Table 3). The two dominant phyla in control animals (*n* = 5), *Firmicutes* (83.36%) and *Bacteroidetes* (20.34%), exhibited dramatic reductions in the dysbiosis group to 5.00% and 1.02%, respectively. This represented an approximate 94% reduction in *Firmicutes* abundance and 95% reduction in *Bacteroidetes* abundance, confirming successful dysbiosis induction.

**TABLE 3:** Percentage of 16S Taxon‐Specific Copy Numbers for Different Phyla between Control and Antibiotics treatment group 14 days posttreatment.

Phylum	Percentage of 16S taxon‐specific copy number in control group	Log Value in control group	Percentage of 16S taxon‐specific copy number in antibiotics treated group	Log Value in antibiotics group
*Bacteroidetes*	20.34	3.01	1.02	0.02
*Firmicutes*	83.36	4.42	5.00	1.61
*Deferribacteres*	1.00	0.00	1.00	0.00
*Candidatus Saccharibacteria*	1.00	0.00	1.00	0.00
*Actinobacteria*	1.12	0.11	1.00	0.00
*Tenericutes*	1.00	0.00	1.00	0.00
*Protobacteria*	1.20	0.18	1.00	0.00
*Verrucomicrobia*	1.00	0.00	1.00	0.00

Minor phyla, including *Deferribacteres*, *Candidatus* Saccharibacteria, *Actinobacteria*, *Tenericutes*, *Proteobacteria*, and *Verrucomicrobia*, showed minimal changes, maintaining relatively stable abundances between 1.00% and 1.20% across both groups. In contrast, major shifts occurred in the dominant phyla, with log‐transformed abundance data revealing the magnitude of these changes. The *Firmicutes*/*Bacteroidetes* ratio, a key indicator of gut microbiome health, shifted from 4.10 in controls to 4.90 in dysbiotic animals, indicating severe microbial imbalance. However, the log‐value analysis provided deeper insight into the underlying microbial dynamics: *Firmicutes* abundance showed a significant 2.8‐fold logarithmic decrease (from 4.42 to 1.61), while *Bacteroidetes* exhibited a more dramatic 3.0‐fold logarithmic reduction (from 3.01 to 0.02) in dysbiotic animals. These substantial log‐scale reductions indicate not merely proportional changes but exponential losses in microbial populations, suggesting profound disruption of the gut ecosystem's structural integrity and functional capacity.

### 3.2 Behavioral Consequences of Gut Dysbiosis

#### 3.2.1 Spatial Working Memory Impairment

**FIGURE 1:**
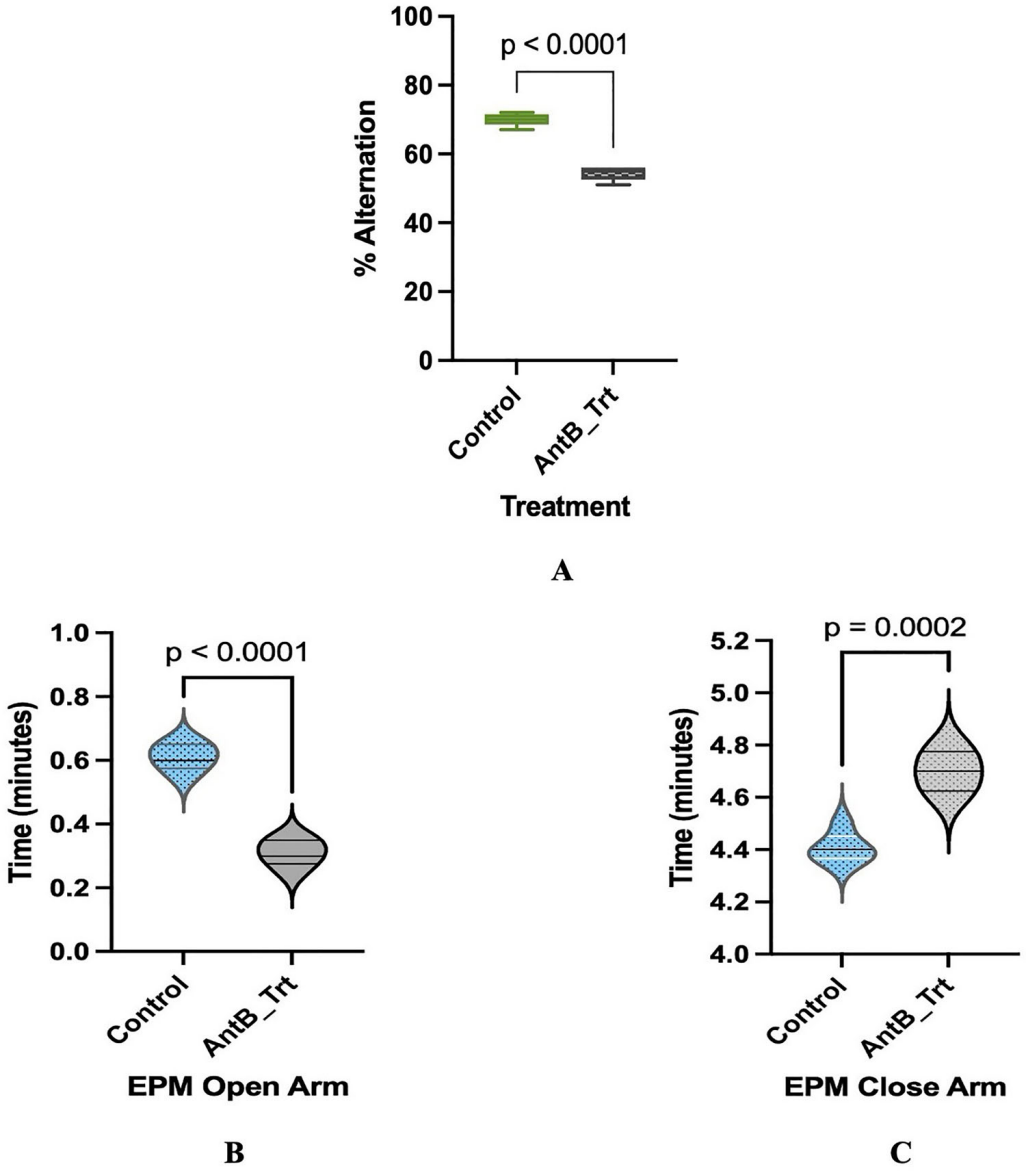
Illustrates cognitive and anxiety‐related behavioral impairments in dysbiotic mice. (A) Antibiotic treatment significantly impairs spontaneous alternation behavior in the Y‐maze test compared to controls. Dysbiotic mice show reduced spatial working memory, with data presented as mean ± SEM. Statistical analysis (unpaired *t*‐test, *n* = 5 per group) reveals a highly significant difference (*p* < 0.0001). (B) Assessment of anxiety‐related behavior on the Elevated Plus Maze shows that dysbiotic animals spend significantly less time exploring open arms compared to controls, indicating increased anxiety‐like behavior (*n* = 5 per group; *p* < 0.001). (C) Dysbiotic mice demonstrate a marked preference for closed arms, spending more time in protected areas than controls, further supporting an anxiety‐like phenotype (*n* = 5 per group; *p* < 0.0002).

Y‐maze spontaneous alternation analysis revealed significant cognitive deficits in dysbiotic mice (Figure 1A). Control animals (*n* = 5) achieved 70% correct alternations, consistent with normal spatial working memory function. In contrast, dysbiotic mice demonstrated significantly impaired performance with only 56% correct alternations (*p* < 0.0001, *t* = 12.49, df = 8). The observed reduction in alternation performance indicates substantial working memory dysfunction following gut microbiome disruption (Figure 1A).

#### 3.2.2 Anxiety‐Like Behavioral Phenotype

The elevated plus maze assessment demonstrated that gut dysbiosis significantly increased anxiety‐like behaviors in mice (Figure 1B,C). Dysbiotic animals (*n* = 5) exhibited a marked avoidance of the anxiety‐provoking open arms, spending only half as much time exploring these areas compared to controls (Figure 1B) (*n* = 5, 18 vs. 36 s; *p* < 0.0001, *t* = 11.34, df = 8). This anxious phenotype was further confirmed by the reciprocal pattern in closed arm exploration, where dysbiotic mice showed significantly longer dwelling times in the safer enclosed areas (Figure 1C) (282 vs. 264 s; *p* = 0.0002, *t* = 6.770, df = 7). These behavioral changes indicate that disruption of the gut microbiome produces a robust anxiety‐like state that drives animals to seek sheltered environments while avoiding open, exposed spaces.

#### 3.2.3 Locomotor Activity Assessment

**FIGURE 2:**
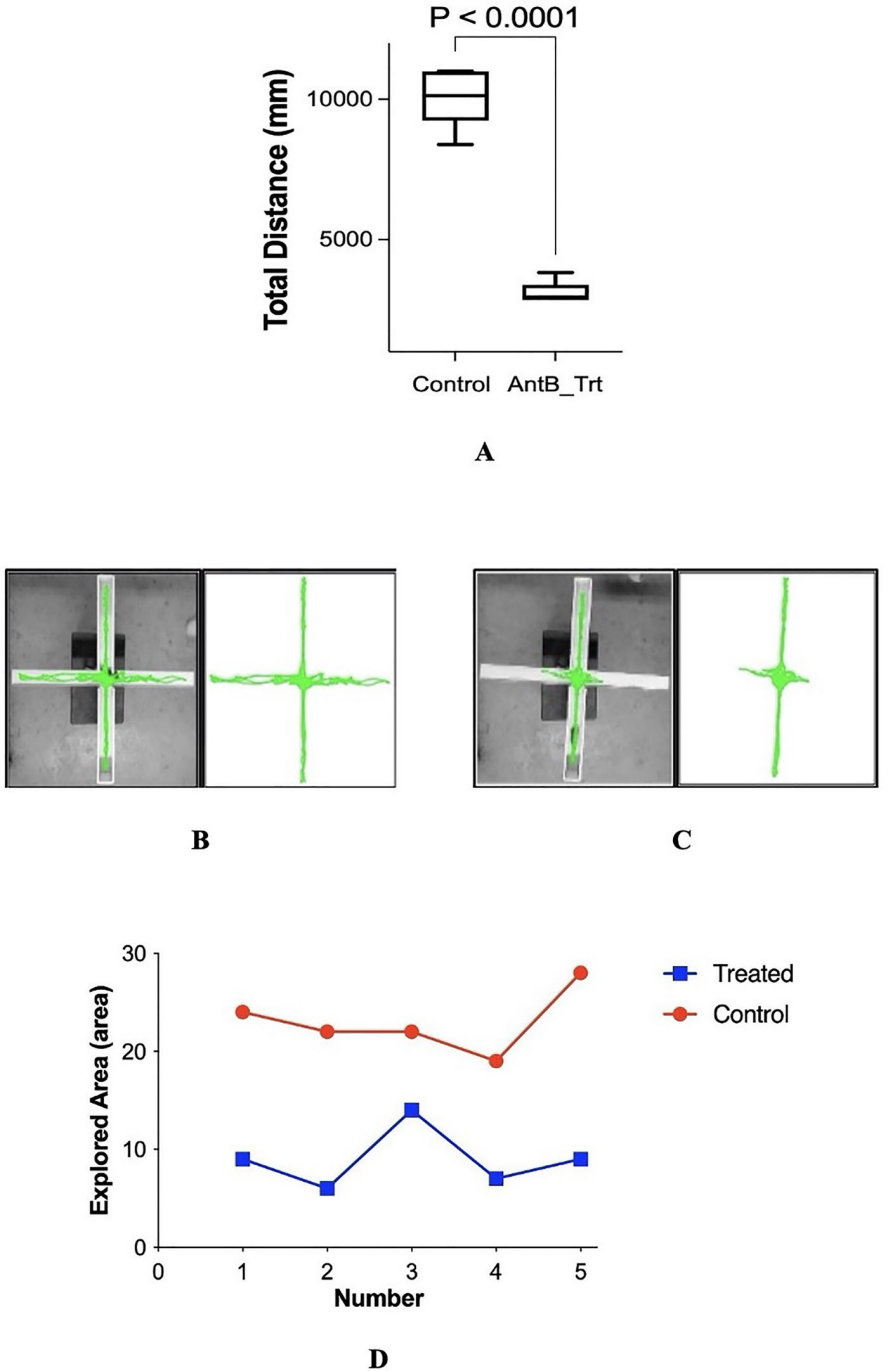
Demonstrates reduced locomotor activity and exploration in dysbiotic mice on the Elevated Plus Maze. (A) Total distance traveled on the Elevated Plus Maze. Control mice showed significantly greater locomotor activity, covering about four times the distance of dysbiotic mice during the 5‐min test (*n* = 5/group; mean ± SEM; unpaired *t*‐test, *p* < 0.0001). (B) Representative exploration pattern of the control group. Control animals displayed distributed movement throughout all maze compartments, indicating normal exploratory behavior. (C) Exploration pattern of the dysbiotic group. Dysbiotic mice exhibited restricted movement and reduced spatial investigation, reflecting behavioral changes due to gut dysbiosis. (D) Spatial exploration patterns across EPM areas. Dysbiotic mice consistently explored less across all maze regions compared to controls, demonstrating a pronounced anxiety‐like behavioral phenotype (*n* = 5/group; *p* = 0.0001).

Analysis of total distance traveled on the EPM revealed significant variations in locomotor activity between experimental groups. The control group covered substantially greater total distance (*n* = 5, 10,120 millimeter [mm]) compared to the dysbiotic group (*n* = 5, 3084 mm), traveling approximately fourfold greater distance throughout the testing period (*p* < 0.0001, *t* = 13.78, df = 8; Figure 2A).

Specifically, control animals exhibited robust locomotor activity with consistent movement patterns across the maze compartments. In contrast, dysbiotic animals showed markedly reduced overall movement and locomotor activity throughout the EPM apparatus, a change observed following antibiotics‐induced gut microbiota dysbiosis.

The locomotor data indicated that dysbiotic animals spent considerably more time stationary or engaged in minimal movement behaviors compared to control counterparts. Control animals demonstrated sustained mobility throughout the 5‐min testing session, whereas dysbiotic animals showed reduced movement initiation and shorter movement episodes. This approximately 75% reduction in total distance traveled was consistently observed across individual animals within the dysbiotic group, indicating a behavioral phenotype associated with gut microbiome disruption.

Behavioral assessment, as assessed by visual tracking, revealed notable disparities in exploratory patterns between experimental groups. Mice subjected to antibiotics‐induced gut microbiome dysbiosis exhibited markedly reduced overall exploratory behavior compared to control animals (Figure 2B,C). Specifically, these mice demonstrate lower exploration across the maze compartments.

Behavioral assessment revealed significant differences in exploratory patterns between experimental groups. The dysbiotic mice demonstrated a marked decrease in exploratory behavior compared to control animals (Figure 2D). Specifically, these mice exhibited reduced total distance traveled throughout the EPM (*p* < 0.0001, *t* = 6.914, df = 8). This reduction in exploratory behavior was consistently observed across the maze compartments within the dysbiotic group.

### 3.3 Region‐Specific Gene Expression Alterations

#### 3.3.1 AChE Expression

**FIGURE 3:**
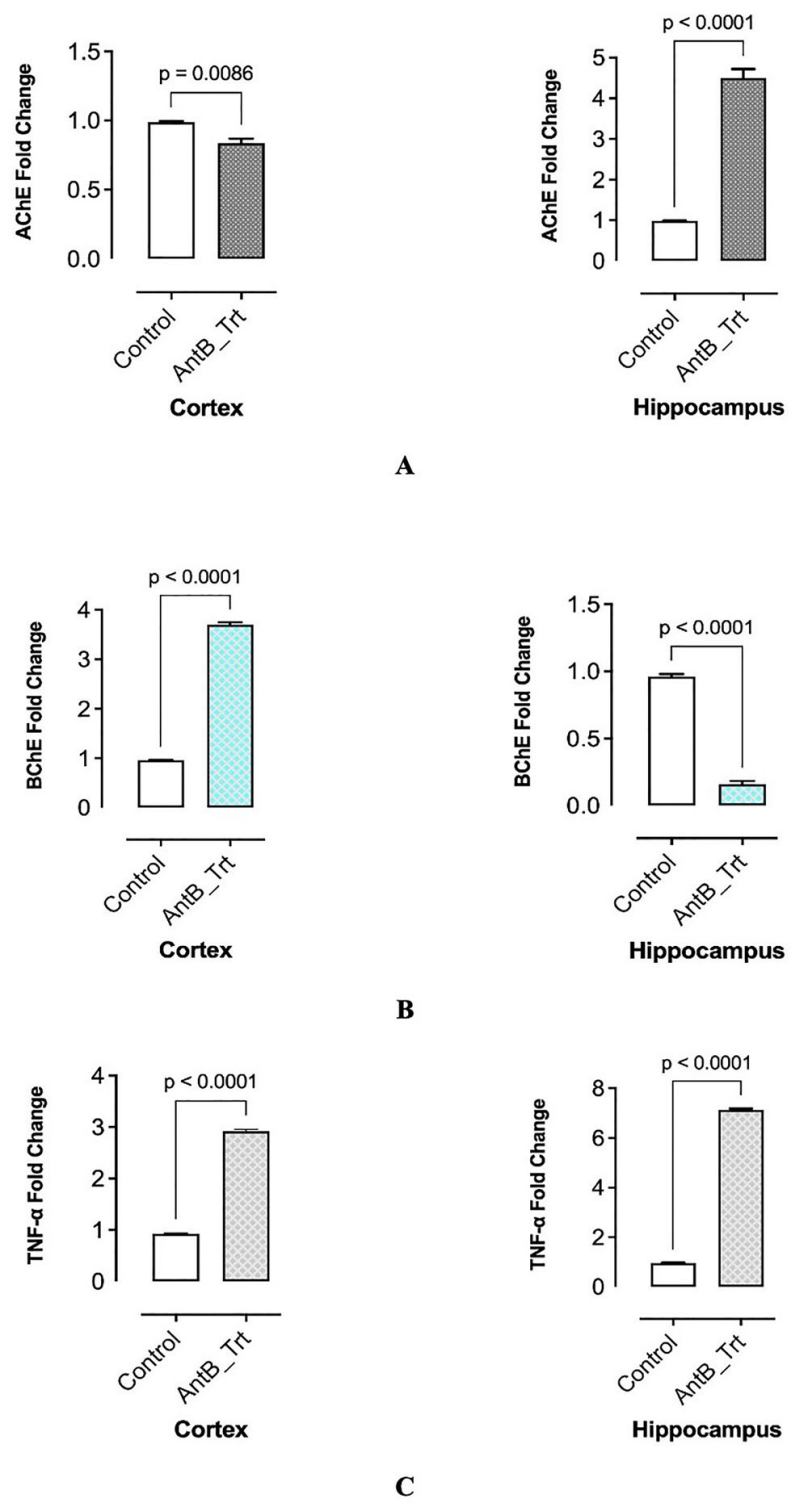
Shows region‐specific cholinergic and inflammatory gene dysregulation in dysbiotic mice. (A) Region‐specific regulation of AChE. Dysbiotic mice exhibit significant downregulation of cortical AChE, while hippocampal AChE is strongly upregulated, implying distinct compensatory responses to gut microbiome disruption. (B) Reciprocal regulation of BChE. Cortical BChE is markedly upregulated, whereas hippocampal BChE is downregulated, highlighting selective cholinergic remodeling after dysbiosis. (C) TNF‐α upregulation. Dysbiotic animals show a 3.1‐fold increase in cortical TNF‐α and an even greater 6.8‐fold increase in hippocampal TNF‐α, indicating heightened susceptibility of the hippocampus to dysbiosis‐induced neuroinflammation.

AChE gene expression exhibited contrasting patterns between brain regions (Figure 3A). In the cortex, dysbiotic mice (*n* = 5) showed significant downregulation of AChE expression relative to controls (*p* = 0.0086, *t* = 4.463, df = 4). Conversely, hippocampal AChE expression was dramatically upregulated in dysbiotic animals (*p* < 0.0001, *t* = 15.71, df = 4). This bidirectional regulation suggests region‐specific compensatory mechanisms in response to gut dysbiosis.

#### 3.3.2 BChE Expression

BChE expression demonstrated an inverse pattern to AChE regulation (Figure 3B). Cortical BChE was significantly upregulated in dysbiotic mice (*n* = 5; 2.8‐fold increase; *p* < 0.0001, *t* = 59.37, df = 4), while hippocampal BChE expression was markedly reduced (*p* < 0.0001, *t* = 26.38, df = 7). This reciprocal regulation pattern between AChE and BChE suggests complex cholinergic system remodeling following gut microbiome disruption.

#### 3.3.3 TNF‐α Expression

TNF‐α expression was consistently upregulated in both brain regions of dysbiotic animals (*n* = 5) (Figure 3C). Cortical TNF‐α showed a 3.1‐fold increase (*p* < 0.0001, *t* = 51.09, df = 4), while hippocampal TNF‐α exhibited an even more pronounced 6.8‐fold elevation (*p* < 0.0001, *t* = 115.0, df = 4). The greater magnitude of hippocampal TNF‐α upregulation suggests this region may be particularly susceptible to dysbiosis‐induced neuroinflammation.

### 3.4 Protein Aggregation Analysis

#### 3.4.1 Amyloid‐β_1–42_ Accumulation

**FIGURE 4:**
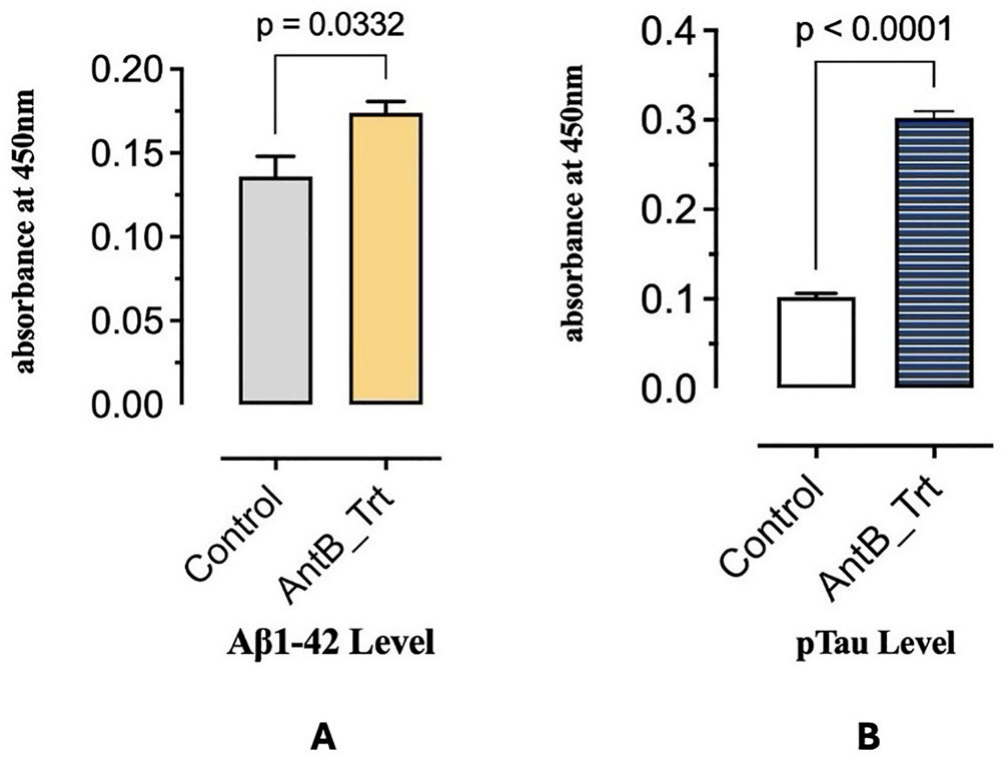
Demonstrates increased amyloid‐β and phosphorylated tau levels in dysbiotic mice. (A) Antibiotic treatment results in a modest but statistically significant increase in amyloid‐β_1–42_ (Aβ_1–42_) levels compared to controls (*n* = 5/group), as measured by ELISA (absorbance at 450 nm; mean ± SEM; unpaired *t*‐test, *p* = 0.0332). (B) Antibiotic treatment significantly elevates phosphorylated tau (pTau) levels relative to controls (*n* = 5/group), as measured by ELISA (absorbance at 450 nm; mean ± SEM; unpaired *t*‐test, *p* < 0.0001).

ELISA quantification revealed significant increases in cerebral Aβ_1–42_ protein levels following gut dysbiosis (Figure 4A). Control mice (*n* = 5) exhibited baseline Aβ_1–42_ concentrations of 0.13 ng/mL protein, while dysbiotic animals (*n* = 5) showed elevated levels of 0.17 ng/mL protein (*p* = 0.0332, *t* = 3.082, df = 4). This increase in Aβ_1–42_ accumulation indicates accelerated amyloid pathology following microbiome disruption.

#### 3.4.2 Total Tau Protein Elevation

Total tau protein analysis demonstrated dramatic increases in dysbiotic mice (Figure 4B). Control animals (*n* = 5) had tau levels of 0.1 ng/mg protein, while dysbiotic mice (*n* = 5) exhibited a striking 3‐fold elevation to 0.3 ng/mg protein (*p* < 0.0001, *t* = 23.64, df = 6). This substantial increase in tau accumulation suggests accelerated tauopathy development following gut microbiome dysbiosis.

## 4 Discussion

The study's finding of a dramatic 94%–95% reduction in *Firmicutes* and *Bacteroidetes* following antibiotic treatment aligns perfectly with current research. Studies have found that dysbiosis in gut microbiota is linked to AD pathophysiological changes, such as abnormal brain Aβ aggregation, inflammation response, immune dysfunction, and neuronal and synaptic damage, with significant alterations in the gut microbial composition observed in AD, showing increased pro‐inflammatory bacteria and decreased anti‐inflammatory bacteria (Liang et al. 2024; D'Argenio et al. 2022; Medeiros et al. 2024). This study demonstrates successful dysbiosis induction, providing a robust experimental model that addresses a critical gap in the field. Recent work has proposed that disruption of the normal gut microbiome in early life leads to a pathological cascade within septohippocampal and cortical brain circuits (Ginsberg and Blaser 2024), and this study extends this concept to demonstrate how acute dysbiosis in aged mice with established AD pathology can accelerate disease progression.

The three‐fold cortical and seven‐fold hippocampal increase in TNF‐α expression observed in this study provides mechanistic validation for the gut–brain inflammatory axis in AD. This finding strongly supports recent studies emphasizing that gut microbiota modulates neuroinflammation in AD through crucial factors and mechanistic underpinnings (Yang et al. 2024; Y. Zhang et al. 2021). The regional specificity of TNF‐α upregulation, with the hippocampus showing greater vulnerability, corroborates clinical observations where the hippocampus is particularly affected in AD progression. There is strong increasing evidence supporting a role for the gut microbiome in the pathogenesis of AD, including effects on synaptic dysfunction and neuroinflammation, which contribute to cognitive decline (Kowalski and Mulak 2019), while this study provides further data in support of the causal impact of gut microbiome dysbiosis in exacerbating disease pathology.

The study's most novel contribution lies in demonstrating region‐specific, reciprocal regulation of cholinergic enzymes following gut dysbiosis. The cortical AChE downregulation with concurrent hippocampal upregulation, paired with opposite BChE patterns, reveals a previously uncharacterized mechanism linking gut health to cognitive function. This finding fills a significant knowledge gap, as regulating the gut flora ecological balance upregulates neurotrophic factor expression (Zou et al. 2024). The reciprocal cholinergic enzyme regulation suggests sophisticated compensatory mechanisms that may initially maintain cognitive function but ultimately contribute to dysfunction when dysbiosis persists.

The observed increase in Aβ_1–42_ and three‐fold elevation in total tau protein directly support the causal relationship between gut dysbiosis and AD pathological hallmarks. The gut microbiome has been shown to potentially regulate neuroinflammation in a variety of neurological conditions, including Multiple Sclerosis, Parkinson's disease, and AD (S. Zhang et al. 2024). These protein aggregation findings are particularly significant because they demonstrate that gut dysbiosis doesn't merely correlate with AD pathology but actively accelerates it.

The reduction observed in Y‐maze alternation performance and marked anxiety‐like behaviors in this study provides functional validation of the molecular changes observed. Recent studies have explored the relationships between gut microbiota and specific cognitive domains of AD patients (Chen et al. 2024), and this work provides experimental evidence for these clinical observations. The observed reduction in locomotor activity represents a particularly striking finding that may reflect the systemic impact of gut dysbiosis on brain function beyond traditional cognitive measures.

Our findings provide an interesting counterpoint to those of Hao et al. (2024), highlighting the complex, context‐dependent influence of the gut microbiome on AD pathogenesis. In our study, broad‐spectrum antibiotic‐induced gut dysbiosis in aged (46–48 weeks) 3xTg‐AD mice precipitated marked cognitive decline, pronounced neuroinflammatory responses—most notably, increased TNF‐α expression—and accelerated accumulation of amyloid‐β and tau. This sharply contrasts with the results of Hao et al. (2024), who reported that gut bacterial depletion in young (3–5 months) APP‐transgenic mice attenuated neuroinflammation and reduced amyloid burden through mechanisms involving IL‐17a signaling.

Several critical factors may underlie these divergent outcomes. Foremost among them is the age and disease stage of the experimental animals. While Hao et al. demonstrated that antibiotic‐mediated microbiota depletion confers neuroprotection in young AD mice, they also observed that the same intervention increased neuroinflammatory gene expression in aged (24‐month‐old) wild‐type animals. Our data, derived from late‐stage AD mice, reinforce the notion that the aged brain is particularly vulnerable to the deleterious effects of gut dysbiosis—potentially reflecting immunosenescence and the amplification of systemic inflammatory signals in the context of advanced neurodegeneration (Colombo et al. 2021; Erny et al. 2015).

Additionally, differences in genetic background and disease trajectory (3xTg‐AD vs. APP/PS1) may further modulate the interplay between gut microbiota and central nervous system pathology, influencing both the baseline immune milieu and the response to microbial perturbation (Harach et al. 2017; Minter et al. 2017).

Our results also indicate that gut dysbiosis in aged mice elicits robust neuroinflammatory responses, as evidenced by pronounced TNF‐α upregulation in both hippocampus and cortex, and accelerates the aggregation of pathological proteins. By contrast, Hao et al. found that gut bacterial depletion in young mice suppressed microglial activation and reduced IL‐17a‐expressing CD4+ T cells, thereby conferring neuroprotection. These findings suggest that the immune landscape of the aged brain is fundamentally altered, resulting in heightened sensitivity to systemic inflammatory cues from the gut (Hao et al. 2024; Colombo et al. 2021).

Finally, the resilience and regenerative capacity of the gut microbiome itself diminish with age, raising the possibility that antibiotic‐induced dysbiosis in older animals leads to persistent microbial imbalance and chronic immune dysregulation (Erny et al. 2015).

Collectively, these observations underscore the critical importance of age, disease stage, and host immune status in determining the neurobiological consequences of gut microbiome modulation. Therapeutic strategies targeting the gut–brain axis in AD must therefore be tailored to the individual's age and disease context, and future studies should systematically address these variables to inform the design of precision microbiome‐based interventions (Hao et al. 2024; Harach et al. 2017; Minter et al. 2017; Colombo et al. 2021; Erny et al. 2015).

The study's results strongly support recent therapeutic directions in the field. Recent research on pharmacogenomics and the gut microbiota has provided a platform for developing novel approaches to treat AD, with modulation of gut microbiota through drug interventions offering a promising method of treating AD. Current research is exploring gut microbiota metabolites as potential therapeutic targets for AD (Bairamian et al. 2022), and this study provides the mechanistic foundation supporting such approaches by demonstrating a causal link between dysbiosis and AD progression.

### 4.1 Study Limitations and Future Directions

Despite the robust findings presented, several limitations of this study must be acknowledged. First, the small sample size (*n* = 5 per group) may restrict the statistical power and generalizability of the results. Second, the exclusive use of female 3xTg‐AD mice, although justified by their more pronounced AD pathology and survival rates, precludes assessment of potential sex‐dependent effects on gut–brain axis modulation and disease progression. Additionally, molecular analyses focused on a select panel of cholinergic and inflammatory markers; broader transcriptomic profiling would provide a more comprehensive understanding of the gut–brain axis in AD.

Future studies should aim to increase sample sizes and incorporate both sexes to evaluate potential sex‐specific responses. Multi‐omics approaches—including comprehensive transcriptomic, proteomic, and metabolomic profiling—would further elucidate the mechanistic links between gut microbiota alterations and AD progression. These efforts will be essential to refine our understanding of gut–brain axis dynamics and to inform the development of targeted interventions for AD.

## 5 Conclusion

This study provides evidence that antibiotic‐induced gut dysbiosis accelerates AD pathology in aged 3xTg‐AD mice. Profound microbial disruption led to significant cognitive and behavioral impairments, marked by reduced spatial memory, heightened anxiety‐like behaviors, and diminished locomotor activity. Molecular analyses revealed region‐specific dysregulation of cholinergic enzymes—AChE and BChE—alongside pronounced neuroinflammatory responses characterized by elevated TNF‐α expression. Furthermore, gut dysbiosis significantly increased the accumulation of amyloid‐β_1–42_ and tau proteins, hallmark features of AD progression. These findings strongly suggest a causal mechanistic link between gut microbiome disruption and the exacerbation of AD, highlighting the gut–brain axis as a pivotal modulator of neurodegenerative disease. This work fills a critical gap in the field by demonstrating that acute microbial imbalance in late‐stage AD is sufficient to aggravate neuropathology and behavioral deficits. Ultimately, the study reinforces the therapeutic potential of targeting gut microbiota as a novel intervention strategy in AD.

## Author Contributions

E.J.T., A.O., and M.Y.O.‐A. conceived the study. E.J.T., D.L.S., M.M., E.A.‐B., and S.A. designed and performed the study. E.J.T., A.O., and M.Y.O.‐A. analyzed the study data. All authors contributed to the interpretation of results. E.J.T., D.L.S., M.M., E.A.‐B. and S.A. drafted the manuscript, while A.O. and M.Y.O.‐A. reviewed and edited the draft. All authors read and approved the final manuscript and agreed to be accountable for all aspects of the work.

## Consent

The authors have nothing to report.

## Conflicts of Interest

The authors declare no conflicts of interest.

## Peer Review

The peer review history for this article is available at https://publons.com/publon/10.1002/brb3.70946

## Data Availability

The datasets generated and analyzed during the current study are available from the corresponding author upon reasonable request. No publicly archived datasets were used or generated in this study.

## References

[brb370946-bib-0001] Adamu, A. , S. Li , F. Gao , and G. Xue . 2024. “The Role of Neuroinflammation in Neurodegenerative Diseases: Current Understanding and Future Therapeutic Targets.” Frontiers in Aging Neuroscience 16: 1347987. 10.3389/fnagi.2024.1347987. 38681666 PMC11045904

[brb370946-bib-0002] Ahmad, F. , S. A. Soelar , M. A. Ahmed Adam , M. R. Abu Hassan , and M. A. Yunus . 2025. “MicroRNA Isolation From Urine: Comparison of Total RNA Isolation (TRI) Reagent and Silica Membrane‐Based Extraction Methods.” Clinica Chimica Acta 576: 120396. 10.1016/j.cca.2025.120396. 40441341

[brb370946-bib-0003] Alonso‐García, P. , R. Martín , and E. Martínez‐Pinilla . 2021. “Gut Microbial Imbalance and Neurodegenerative Proteinopathies: From Molecular Mechanisms to Prospects of Clinical Applications.” Exploration of Neuroprotective Therapy 1: 33–54.

[brb370946-bib-0004] Alzheimer, A. 1906. “Uber einen eigenartigen schweren Erkrankungsprozess der Hirninde.” Neurologisches Centralblatt 23: 1129–1136.

[brb370946-bib-0005] Alzheimer, A. , R. A. Stelzmann , H. N. Schnitzlein , and F. R. Murtagh . 1995. “An English Translation of Alzheimer's 1907 Paper, “Uber Eine Eigenartige Erkankung Der Hirnrinde”.” Clinical Anatomy 8: 429–431. 8713166 10.1002/ca.980080612

[brb370946-bib-0006] Anand, N. , V. Gorantla , and S. Chidambaram . 2022. “The Role of Gut Dysbiosis in the Pathophysiology of Neuropsychiatric Disorders.” Cells 12, no. 1: 54. 10.3390/cells12010054. 36611848 PMC9818777

[brb370946-bib-0007] Bairamian, D. , S. Sha , N. Rolhion , et al. 2022. “Microbiota in Neuroinflammation and Synaptic Dysfunction: A Focus on Alzheimer's Disease.” Molecular Neurodegeneration 17: 19. 10.1186/s13024-022-00522-2. 35248147 PMC8898063

[brb370946-bib-0008] Berson, A. , and H. Soreq . 2010. “It All Starts at the Ends: Multifaceted Involvement of C‐ and N‐Terminally Modified Cholinesterases in Alzheimer's Disease.” Rambam Maimonides Medical Journal 1: e0014. 23908786 10.5041/RMMJ.10014 PMC3678781

[brb370946-bib-0009] Bertsch, M. , B. Franchi , M. C. Tesi , and V. Tora . 2023. “The Role of Beta‐Amyloid and Tau Proteins in Alzheimer's Disease: A Mathematical Model on Graph.” Journal of Mathematical Biology 87: 49. 10.1007/s00285-023-01985-7. 37646953 PMC10468937

[brb370946-bib-0010] Bhardwaj, N. , D. Rana , and J. Kaur . 2025. “An Optimized RNA Extraction Method from Micro‐Quantities of Guinea Pig Cartilage and Synovium for Osteoarthritis Research.” Bio‐Protocol Journal 15: e5348. 10.21769/BioProtoc.5348. PMC12222635 40620809

[brb370946-bib-0011] Braak, H. , and E. Braak . 1991. “Neuropathological Stageing of Alzheimer‐Related Changes.” Acta Neuropathologica 82: 239–259. 10.1007/BF00308809. 1759558

[brb370946-bib-0012] Câmara, A. B. 2020. “Alzheimer's Disease Neuroprotection: Associated Receptors.” In Neuroprotection—New Approaches and Prospects, edited by M. Otero‐Losada , F. Capani , and S. P. Lloret . IntechOpen.

[brb370946-bib-0013] Chen, Q. , J. Shi , G. Yu , et al. 2024. “Gut Microbiota Dysbiosis in Patients With Alzheimer's Disease and Correlation With Multiple Cognitive Domains.” Frontiers in Aging Neuroscience 16: 1478557. 10.3389/fnagi.2024.1478557. 39665039 PMC11632125

[brb370946-bib-0014] Colombo, A. V. , R. K. Sadler , G. Llovera , et al. 2021. “Microbiota‐Derived Short Chain Fatty Acids Modulate Microglia and Promote Aβ Plaque Deposition.” eLife 10: e59826. 10.7554/eLife.59826. 33845942 PMC8043748

[brb370946-bib-0015] Coulson, D. T. R. , S. Brockbank , J. G. Quinn , et al. 2008. “Identification of Valid Reference Genes for the Normalization of RT qPCR Gene Expression Data in Human Brain Tissue.” BMC Molecular Biology 9: 46. 10.1186/1471-2199-9-46. 18460208 PMC2396658

[brb370946-bib-0016] Dağdeviren, M. , and E. Bozcal . 2025. “Inflecting Factors on Alzheimer's Disease Progression: The Interaction of Gut Microbiome, Oxidative Stress, and Nutritional Interventions.” *Current Topics in Medicinal Chemistry*, ahead of print, March 18. 10.2174/0115680266342624241127071044. PMC13478946 40108895

[brb370946-bib-0017] D'Argenio, V. et al. 2022. “Gut Microbiome and Mycobiome Alterations in an In Vivo Model of Alzheimer's Disease.” Genes 13, no. 9: 1564. 36140732 10.3390/genes13091564 PMC9498768

[brb370946-bib-0018] Erny, D. , A. L. Hrabě De Angelis , D. Jaitin , et al. 2015. “Host Microbiota Constantly Control Maturation and Function of Microglia in the CNS.” Nature Neuroscience 18: 965–977. 10.1038/nn.4030. 26030851 PMC5528863

[brb370946-bib-0019] Fröhlich, E. E. , A. Farzi , R. Mayerhofer , et al. 2016. “Cognitive Impairment by Antibiotic‐Induced Gut Dysbiosis: Analysis of Gut Microbiota–Brain Communication.” Brain, Behavior, and Immunity 56: 140–155. 10.1016/j.bbi.2016.02.020. 26923630 PMC5014122

[brb370946-bib-0020] Garcia, Y. , and N. Esquivel . 2018. “Comparison of the Response of Male BALB/c and C57BL/6 Mice in Behavioral Tasks to Evaluate Cognitive Function.” Behavioural Sciences 8: 14. 10.3390/bs8010014. PMC5791032 29346276

[brb370946-bib-0021] García‐Cabrerizo, R. , C. Carbia , K. J. O´Riordan , H. Schellekens , and J. F. Cryan . 2020. “Microbiota–Gut–Brain Axis as a Regulator of Reward Processes.” Journal of Neurochemistry 157: 1495–1524. 10.1111/jnc.15284. 33368280

[brb370946-bib-0022] Ginsberg, S. D. , and M. J. Blaser . 2024. “Alzheimer's Disease Has Its Origins in Early Life via a Perturbed Microbiome. Supplement.” Journal of Infectious Diseases 230, no. S2: S141–S149. 10.1093/infdis/jiae200. 39255394 PMC11385592

[brb370946-bib-0023] Goschorska, M. , I. Baranowska‐Bosiacka , I. Gutowska , E. Metryka , M. Skórka‐Majewicz , and D. Chlubek . 2018. “Potential Role of Fluoride in the Etiopathogenesis of Alzheimer's Disease.” International Journal of Molecular Sciences 19: 3965. 10.3390/ijms19123965. 30544885 PMC6320968

[brb370946-bib-0024] Hampel, H. , M.‐M. Mesulam , A. C. Cuello , et al. 2018. “The Cholinergic System in the Pathophysiology and Treatment of Alzheimer's Disease.” Brain 141: 1917–1933. 10.1093/brain/awy132. 29850777 PMC6022632

[brb370946-bib-0025] Hao, W. , Q. Luo , I. Tomic , et al. 2024. “Modulation of Alzheimer's Disease Brain Pathology in Mice by Gut Bacterial Depletion: The Role of IL‐17a.” Gut Microbes 16: 2363014. 10.1080/19490976.2024.2363014. 38904096 PMC11195493

[brb370946-bib-0026] Harach, T. , N. Marungruang , N. Duthilleul , et al. 2017. “Reduction of Abeta Amyloid Pathology in APPPS1 Transgenic Mice in the Absence of Gut Microbiota.” Scientific Reports 7: 41802. 10.1038/srep41802. 28176819 PMC5297247

[brb370946-bib-0027] Harini, V. S. , R. Marimuthu , M. S. A. Tantry , and K. Santhakumar . 2024. “Induction of Paraquat‐Mediated Parkinsonian Phenotype in Zebrafish.” Current Protocols 4: e990. 10.1002/cpz1.990. 38348973

[brb370946-bib-0028] Jabir, N. R. , F. R. Khan , and S. Tabrez . 2018. “Cholinesterase Targeting by Polyphenols: A Therapeutic Approach for the Treatment of Alzheimer's Disease.” CNS Neuroscience & Therapeutics 24: 753–762. 10.1111/cns.12971. 29770579 PMC6489761

[brb370946-bib-0029] Jaszczyk, A. , A. M. Stankiewicz , and G. R. Juszczak . 2022. “Dissection of Mouse Hippocampus With Its Dorsal, Intermediate and Ventral Subdivisions Combined With Molecular Validation.” Brain Sciences 12: 799. 10.3390/brainsci12060799. 35741684 PMC9221087

[brb370946-bib-0030] Judd, J. M. , W. Winslow , I. Mcdonough , F. Mistry , and R. Velazquez . 2024. “Modifying Reaction Time Tasks Parameters in the Automated IntelliCage Identifies Heightened Impulsivity and Impaired Attention in the 3xTg‐AD Model of Alzheimer's Disease.” Frontiers in Aging Neuroscience 16: 1466415. 10.3389/fnagi.2024.1466415. 39744522 PMC11688410

[brb370946-bib-0031] Jürgenson, M. , A. Aonurm‐Helm , and A. Zharkovsky . 2010. “Behavioral Profile of Mice With Impaired Cognition in the Elevated Plus‐Maze due to a Deficiency in Neural Cell Adhesion Molecule.” Pharmacology Biochemistry and Behavior 96: 461–468. 10.1016/j.pbb.2010.07.006. 20624419

[brb370946-bib-0032] Kowalski, K. , and A. Mulak . 2019. “Brain–Gut–Microbiota Axis in Alzheimer's Disease.” Journal of Neurogastroenterology and Motility 25: 48–60. 10.5056/jnm18087. 30646475 PMC6326209

[brb370946-bib-0033] Kraeuter, A. K. , P. C. Guest , and Z. Sarnyai . 2019. “The Y‐Maze for Assessment of Spatial Working and Reference Memory in Mice.” Methods in Molecular Biology 1916: 105–111. 30535688 10.1007/978-1-4939-8994-2_10

[brb370946-bib-0034] Lee, S. , J.‐H. Kwak , S. H. Kim , et al. 2019. “Comparative Study of Liver Injury Induced by High‐Fat Methionine‐ and Choline‐Deficient Diet in ICR Mice Originating From Three Different Sources.” Laboratory Animal Research 35: 15. 10.1186/s42826-019-0016-y. 32257903 PMC7081597

[brb370946-bib-0035] Leo, L. M. , and F. A. Pamplona . 2014. “Elevated Plus Maze Test to Assess Anxiety‐Like Behavior in the Mouse.” Bio‐Protocol 4: e1211. 10.21769/BioProtoc.1211.

[brb370946-bib-0036] Lestaevel, P. , H. Bensoussan , R. Racine , F. Airault , P. Gourmelon , and M. Souidi . 2011. “Transcriptomic Effects of Depleted Uranium on Acetylcholine and Cholesterol Metabolisms in Alzheimer's Disease Model.” Comptes Rendus Biologies 334: 85–90. 10.1016/j.crvi.2010.12.004. 21333939

[brb370946-bib-0037] Liang, Y. , C. Liu , M. Cheng , et al. 2024. “The Link Between Gut Microbiome and Alzheimer's Disease: from the Perspective of New Revised Criteria for Diagnosis and Staging of Alzheimer's Disease.” Alzheimers Dement 20: 5771–5788. 10.1002/alz.14057. 38940631 PMC11350031

[brb370946-bib-0038] Liu, Y.‐S. et al. 2023. “Research Progress on the Etiology and Pathogenesis of Alzheimer's Disease From the Perspective of Chronic Stress.” Aging and Disease 14: 1292–1310. 37163426 10.14336/AD.2022.1211 PMC10389837

[brb370946-bib-0039] Medeiros, D. , K. Mcmurry , M. Pfeiffer , et al. 2024. “Slowing Alzheimer's Disease Progression Through Probiotic Supplementation.” Frontiers in Neuroscience 18: 1309075. 10.3389/fnins.2024.1309075. 38510467 PMC10950931

[brb370946-bib-0040] Minter, M. R. , R. Hinterleitner , M. Meisel , et al. 2017. “Antibiotic‐Induced Perturbations in Microbial Diversity During Post‐Natal Development Alters Amyloid Pathology in an Aged APPSWE/PS1ΔE9 Murine Model of Alzheimer's Disease.” Scientific Reports 7: 10411. 10.1038/s41598-017-11047-w. 28874832 PMC5585265

[brb370946-bib-0041] Muntsant, A. , F. Jiménez‐Altayó , L. Puertas‐Umbert , E. Jiménez‐Xarrie , E. Vila , and L. Giménez‐Llort . 2021. “Sex‐Dependent End‐of‐Life Mental and Vascular Scenarios for Compensatory Mechanisms in Mice With Normal and AD‐Neurodegenerative Aging.” Biomedicines 9: 111. 10.3390/biomedicines9020111. 33498895 PMC7911097

[brb370946-bib-0042] Nasb, M. , W. Tao , and N. Chen . 2023. “Alzheimer's Disease Puzzle: Delving Into Pathogenesis Hypotheses.” Aging and Disease 15: 43–73. 10.14336/AD.2023.0608 PMC10796101 37450931

[brb370946-bib-0043] Nguyen, N. M. , J. Cho , and C. Lee . 2023. “Gut Microbiota and Alzheimer's Disease: How to Study and Apply Their Relationship.” International Journal of Molecular Sciences 24: 4047. 10.3390/ijms24044047. 36835459 PMC9958597

[brb370946-bib-0044] Oddo, S. , A. Caccamo , J. D. Shepherd , et al. 2003. “Triple‐Transgenic Model of Alzheimer's Disease With Plaques and Tangles: Intracellular Abeta and Synaptic Dysfunction.” Neuron 39: 409–421. 10.1016/S0896-6273(03)00434-3. 12895417

[brb370946-bib-0045] Özgür, D. et al. 2023. “Investigation of Efflux Pump Genes in Resistant Mycobacterium Tuberculosis Complex Clinical Isolates Exposed to First Line Antituberculosis Drugs and Verapamil Combination.” Mikrobiyoloji bulteni 57: 207–219. 37067206 10.5578/mb.20239916

[brb370946-bib-0046] Rhodes, M. E. , J. S. Kennell , E. E. Belz , R. K. Czambel , and R. T. Rubin . 2004. “Rat Estrous Cycle Influences the Sexual Diergism of HPA Axis Stimulation by Nicotine.” Brain Research Bulletin 64: 205–213. 10.1016/j.brainresbull.2004.06.011. 15464856

[brb370946-bib-0047] Rodriguez, A. , H. Zhang , J. Klaminder , T. Brodin , P. L. Andersson , and M. Andersson . 2018. “ *ToxTrac*: A Fast and Robust Software for Tracking Organisms.” Methods in Ecology and Evolution 9: 460–464. 10.1111/2041-210X.12874.

[brb370946-bib-0048] Sheng, C. , W. Du , Y. Liang , et al. 2023. “An Integrated Neuroimaging‐Omics Approach for the Gut–Brain Communication Pathways in Alzheimer's Disease.” Frontiers in Aging Neuroscience 15: 1211979. 10.3389/fnagi.2023.1211979. 37869373 PMC10587434

[brb370946-bib-0049] Souto‐Maior, F. N. , F. L. de Carvalho , L. C. S. Lima de Morais , S. M. Netto , D. P. De Sousa , and R. N. de Almeida . 2011. “Anxiolytic‐Like Effects of Inhaled Linalool Oxide in Experimental Mouse Anxiety Models.” Pharmacology Biochemistry and Behavior 100: 259–263. 10.1016/j.pbb.2011.08.029. 21925533

[brb370946-bib-0050] Spinelli, R. , I. Sanchís , and A. Siano . 2025. “Fighting Alzheimer's Naturally: Peptides as Multitarget Drug Leads.” Bioorganic & medicinal chemistry letters 127: 130305. 40494420 10.1016/j.bmcl.2025.130305

[brb370946-bib-0051] Torres‐Lista, V. , M. De la Fuente , and L. Giménez‐Llort . 2017. “Survival Curves and Behavioral Profiles of Female 3xTg‐AD Mice Surviving to 18‐Months of Age as Compared to Mice With Normal Aging.” Journal of Alzheimer's Disease Reports 1: 47–57. 10.3233/ADR-170011. PMC6159713 30480229

[brb370946-bib-0052] Wang, P. , K. Tu , P. Cao , et al. 2021. “Antibiotics‐Induced Intestinal Dysbacteriosis Caused Behavioral Alternations and Neuronal Activation in Different Brain Regions in Mice.” Molecular Brain 14: 49. 10.1186/s13041-021-00759-w. 33676528 PMC7937204

[brb370946-bib-0053] Wang, X. , C. Zhao , G. Zhang , et al. 2022. “Molecular Characterization of a Novel GSTO2 of Fasciola Hepatica and Its Roles in Modulating Murine Macrophages.” Parasite 29: 16. 10.1051/parasite/2022016. 35315767 PMC8939299

[brb370946-bib-0054] Xing, H. et al. 2025. “Recent Advances in Drug Development for Alzheimer's Disease: A Comprehensive Review.” International Journal of Molecular Sciences 26: 3905. 40332804 10.3390/ijms26083905 PMC12028297

[brb370946-bib-0055] Yadav, S. , A. Kumar , M. Gupta , and S. S. Maitra . 2018. “Cross‐Reactivity of Prokaryotic 16S rDNA‐Specific Primers to Eukaryotic DNA: Mistaken Microbial Community Profiling in Environmental Samples.” Current Microbiology 75: 1038–1045. 10.1007/s00284-018-1482-4. 29610942

[brb370946-bib-0056] Yang, J. , J. Liang , N. Hu , et al. 2024. “The Gut Microbiota Modulates Neuroinflammation in Alzheimer's Disease: Elucidating Crucial Factors and Mechanistic Underpinnings.” CNS neuroscience & therapeutics 30: e70091. 10.1111/cns.70091. 39460538 PMC11512114

[brb370946-bib-0057] Yang, Y.‐W. , M.‐K. Chen , B.‐Y. Yang , et al. 2015. “Use of 16S rRNA Gene‐Targeted Group‐Specific Primers for Real‐Time PCR Analysis of Predominant Bacteria in Mouse Feces.” Applied and Environmental Microbiology 81: 6749–6756. 10.1128/AEM.01906-15. 26187967 PMC4561689

[brb370946-bib-0058] Zhang, S. , J. Lu , Z. Jin , et al. 2024. “Gut Microbiota Metabolites: Potential Therapeutic Targets for Alzheimer's Disease?” Frontiers in Pharmacology 15: 1459655. 10.3389/fphar.2024.1459655. 39355779 PMC11442227

[brb370946-bib-0059] Zhang, Y. , R. Geng , and Q. Tu . 2021. “Gut Microbial Involvement in Alzheimer's Disease Pathogenesis.” Aging 13: 13359–13371. 10.18632/aging.202994. 33971619 PMC8148443

[brb370946-bib-0060] Zheng, Y. , L. Bonfili , T. Wei , and A. M. Eleuteri . 2023. “Understanding the Gut‐Brain Axis and Its Therapeutic Implications for Neurodegenerative Disorders.” Nutrients 15: 4631. 10.3390/nu15214631. 37960284 PMC10648099

[brb370946-bib-0061] Zou, X. , G. Zou , X. Zou , K. Wang , and Z. Chen . 2024. “Gut Microbiota and Its Metabolites in Alzheimer's Disease: From Pathogenesis to Treatment.” PeerJ 12: e17061. 10.7717/peerj.17061. 38495755 PMC10944166

